# Traditional Chinese medicine reprograms the tumor microenvironment to overcome EGFR-TKI resistance: mechanisms and therapeutic perspectives

**DOI:** 10.3389/fphar.2026.1901815

**Published:** 2026-09-11

**Authors:** Yuan Yao, Shan Gao, Jun-Ping Wang

**Affiliations:** 1 Pharmacy Center, Hefei Cancer Hospital, Chinese Academy of Sciences, Hefei, China; 2 Department of Pharmacology, Basic Medical College, Anhui Medical University, Hefei, China

**Keywords:** drug resistance reversal, EGFR tyrosine kinase inhibitor resistance, non-small cell lung cancer, traditional Chinese medicine, tumor microenvironment

## Abstract

Epidermal growth factor receptor tyrosine kinase inhibitors (EGFR-TKIs) have transformed the treatment of EGFR-mutant non-small cell lung cancer (NSCLC). However, the inevitable emergence of acquired resistance significantly limits their long-term efficacy. While conventional accounts of resistance have primarily focused on tumor-intrinsic genetic alterations, accumulating evidence highlights the pivotal role of the tumor microenvironment (TME) in mediating therapeutic failure. The TME contributes to EGFR-TKI resistance through multiple interconnected mechanisms, including immunosuppression, extracellular matrix-mediated physical barriers, metabolic reprogramming, and compensatory signaling activation. These complex interactions create a protective niche that enables tumor cells to persist despite EGFR inhibition. Traditional Chinese medicine (TCM), characterized by its multi-component and multi-target properties, offers a potential systems-level approach to modulating the TME. Preclinical studies suggest that TCM-derived interventions may influence immune-cell polarization, stromal and vascular features, and tumor metabolism, thereby affecting EGFR-TKI sensitivity. This review focuses on TME-mediated EGFR-TKI resistance in NSCLC and appraises TCM interventions according to evidence type and directness. We evaluate current preclinical and clinical evidence, identify major limitations, and propose priorities for translational research. The review provides a conceptual framework for evaluating TCM-based combination strategies intended to attenuate EGFR-TKI resistance.

## 1 Introduction

Epidermal growth factor receptor tyrosine kinase inhibitors (EGFR-TKIs) have fundamentally transformed the treatment of EGFR-mutant non-small cell lung cancer (NSCLC). These agents have produced substantial improvements in progression-free survival, and osimertinib has also improved overall survival, establishing genotype-directed therapy as the standard approach for this molecular subgroup (Wu et al., 2017; Ramalingam et al., 2020; Passaro et al., 2021).

Despite this initial benefit, acquired resistance develops in most patients and is biologically heterogeneous, ultimately leading to treatment failure (Suda et al., 2009).

Acquired resistance is a general limitation of targeted therapy and is not unique to lung cancer. We selected EGFR-mutant NSCLC as the principal model because it provides a clinically traceable sequence from a defined oncogenic driver through treatment response to relapse. Successive generations of EGFR-TKIs, together with repeat tissue biopsy and plasma genotyping, have generated one of the most detailed longitudinal accounts of targeted-therapy resistance (Oxnard et al., 2018; Passaro et al., 2021). The emphasis on EGFR does not imply that it is the preferred target in all cases of NSCLC. Alterations involving other oncogenic receptor tyrosine kinases (RTKs), including anaplastic lymphoma kinase (ALK), ROS proto-oncogene 1 (ROS1), RET proto-oncogene (RET), and MET receptor tyrosine kinase (MET), define distinct molecular subgroups that require genotype-matched therapy. EGFR is emphasized because its sequential treatment history and resistance spectrum—including on-target mutations, bypass signaling, lineage plasticity, and TME-mediated adaptation—are particularly well characterized. MET and human epidermal growth factor receptor 2 (HER2) are also relevant because they can provide bypass signaling after EGFR inhibition (Passaro et al., 2021; Tan and Tan, 2022).

Mechanistic studies initially focused on tumor-intrinsic resistance, including secondary mutations in target genes (e.g., EGFR T790M and C797S), activation of alternative signaling pathways (such as MET and HER2 amplification), and sustained activation of downstream pathways (Wu and Shih, 2018; He et al., 2021; Engelman et al., 2007). While these mechanisms have been extensively characterized and have led to the development of next-generation inhibitors, they do not fully account for the complexity of resistance observed in clinical settings.

In recent years, the conceptual framework of cancer biology has evolved from a tumor cell–centric view to a more holistic perspective that considers the tumor as a complex ecosystem. Within this context, the TME has emerged as a critical determinant of therapeutic response. The TME consists of a heterogeneous population of immune cells, cancer-associated fibroblasts (CAFs), endothelial cells, extracellular matrix (ECM) components, and a variety of cytokines and metabolic products. Rather than serving as a passive scaffold, the TME actively regulates tumor growth, metastasis, immune evasion, and drug resistance (Binnewies et al., 2018; Hinshaw and Shevde, 2019).

Mechanistically, the TME contributes to EGFR-TKI resistance through four major pathways. First, it establishes an immunosuppressive network that impairs antitumor immune responses. Second, it forms a dense extracellular matrix that acts as a physical barrier, limiting drug penetration into tumor tissues. Third, it induces metabolic reprogramming, leading to hypoxia and an acidic microenvironment that reduces drug efficacy. Fourth, it activates compensatory signaling pathways through paracrine interactions, thereby maintaining tumor cell survival despite effective kinase inhibition (Binnewies et al., 2018).

Because these processes can reinforce one another, targeting a single pathway is often insufficient to reverse established EGFR-TKI resistance. TCM formulations are proposed as multi-component adjuncts capable of modulating several TME compartments, but this systems-level rationale requires disease- and drug-specific experimental validation (Zhao et al., 2023; Liu et al., 2024; Li and Xiao, 2025).

In this review, we examine tumor-intrinsic and TME-mediated mechanisms of EGFR-TKI resistance in NSCLC, critically assess the evidence that TCM interventions can modify these processes, and discuss the requirements for translating TCM-EGFR-TKI combinations into biomarker-guided clinical strategies.

## 2 Mechanisms of TKI resistance

EGFR-TKI resistance may be present before treatment (primary resistance) or emerge after an initial response (acquired resistance), and it reflects the interaction of tumor-intrinsic alterations with adaptive changes in the TME. Tumor-intrinsic mechanisms include secondary EGFR mutations, activation of bypass pathways, downstream signaling reactivation, phenotypic plasticity, evasion of apoptosis, and enhanced drug efflux (Passaro et al., 2021; Wu and Shih, 2018). In parallel, stromal remodeling, recruitment of immunosuppressive cells, hypoxia, and metabolic adaptation may sustain survival signaling or reduce effective EGFR-TKI exposure.

### 2.1 Tumor-intrinsic mechanisms of EGFR-TKI resistance

Tumor-intrinsic resistance to EGFR inhibition arises from genetic, epigenetic, and phenotypic alterations within cancer cells. These mechanisms include secondary mutations in target genes, activation of bypass signaling pathways, dysregulation of downstream signaling cascades, phenotypic plasticity, and alterations in apoptosis and drug efflux.

#### 2.1.1 Secondary EGFR mutations

Secondary mutations in target genes represent the most direct and prevalent mechanism of acquired resistance. These mutations alter the structure of the kinase domain, thereby reducing drug binding affinity and impairing inhibitor efficacy. In EGFR-mutant NSCLC, the substitution of threonine with methionine at position 790 (T790M) is the most well-characterized resistance mutation, accounting for approximately 50% of acquired resistance cases (Wu et al., 2023). This mutation enhances adenosine triphosphate (ATP) affinity, thereby diminishing the competitive inhibitory effect of first-generation TKIs.

Third-generation EGFR inhibitors, such as osimertinib, have improved outcomes in patients harboring T790M mutations. Resistance to osimertinib can involve tertiary EGFR mutations such as C797S, which disrupts covalent binding of the drug to the kinase domain (Kashima et al., 2020; Niederst et al., 2015). Other mutations, including EGFR G724S, have also been reported after osimertinib exposure (Oztan et al., 2017). These findings highlight the dynamic and evolving nature of tumor adaptation under therapeutic pressure.

#### 2.1.2 Activation of bypass signaling pathways

In response to inhibition of primary oncogenic drivers, tumor cells can activate alternative signaling pathways to maintain survival and proliferation. This process, known as bypass signaling, represents a major compensatory mechanism of resistance.

Amplification of the MET proto-oncogene is one of the most common bypass mechanisms, occurring in approximately 10%–20% of patients with acquired resistance to EGFR-TKIs (Sakamoto and Patil, 2023). MET activation leads to downstream signaling through the phosphoinositide 3-kinase/protein kinase B (PI3K/AKT) and mitogen-activated protein kinase (MAPK) pathways, effectively circumventing EGFR inhibition. Similarly, amplification or overexpression of HER2 and AXL receptor tyrosine kinase (AXL) can promote resistance by forming heterodimers or directly activating downstream pathways (Cheng, 2024; Yadav et al., 2025).

These bypass mechanisms underscore the redundancy and plasticity of oncogenic signaling networks, which allow tumor cells to rapidly adapt to targeted therapy.

#### 2.1.3 Dysregulation of downstream signaling pathways

Mutations in downstream signaling components can render tumor cells independent of upstream receptor activity. For instance, activating mutations in KRAS or BRAF result in constitutive activation of the MAPK pathway, thereby sustaining cell proliferation despite effective inhibition of upstream kinases (Passaro et al., 2021; Wu and Shih, 2018).

Similarly, alterations in the PI3K/AKT/mechanistic target of rapamycin (mTOR) pathway, such as PIK3CA mutations or PTEN loss, lead to enhanced cell survival and resistance to apoptosis (Sakamoto and Patil, 2023). These downstream alterations represent a critical mechanism by which tumor cells bypass targeted inhibition and maintain malignant behavior.

#### 2.1.4 Phenotypic plasticity and lineage transformation

Tumor cells can adapt to therapeutic stress through reversible changes in cellular state and identity. Epithelial-to-mesenchymal transition (EMT) is one of the best-characterized forms of such plasticity and is associated with enhanced invasiveness, stem-like properties, and resistance to multiple therapies, including TKIs (Fischer et al., 2015). Importantly, this plasticity is not simply a pre-existing feature that is passively selected during treatment. In EGFR-mutant NSCLC, prolonged EGFR-TKI exposure can induce or stabilize a drug-tolerant mesenchymal state through transcriptional and epigenetic reprogramming involving transforming growth factor beta (TGF-β)/SMAD, nuclear factor kappa-light-chain-enhancer of activated B cells (NF-κB), and AXL-associated signaling. Compared with epithelial cells, these mesenchymal-like cells are less dependent on EGFR signaling and less susceptible to TKI-induced apoptosis. Because this state may initially be reversible, it can provide a reservoir from which stable resistance mechanisms subsequently emerge (Dela Cruz and Medina, 2025). ZEB1-associated loss of EMT-regulating microRNAs has also been reported in EGFR-TKI-resistant NSCLC (Gohlke et al., 2023).

Lineage transformation represents a more extensive form of therapy-associated plasticity. Studies have shown that EGFR-mutant lung adenocarcinoma can undergo transformation into small-cell lung cancer following EGFR-TKI treatment. Transformed tumors frequently retain the original EGFR mutation, supporting a common clonal origin, whereas concurrent inactivation of RB1 and TP53 appears to create a permissive background for neuroendocrine reprogramming (Li et al., 2024; Offin et al., 2019). Thus, TKI treatment acts not only as a selective pressure that enriches pre-existing resistant subpopulations but also as a stimulus that can promote adaptive state transitions and lineage switching, thereby reducing tumor dependence on the originally targeted kinase.

#### 2.1.5 Apoptosis evasion and drug efflux

Alterations in apoptotic signaling and drug transport further contribute to TKI resistance. In oncogene-addicted tumor cells, TKI-induced apoptosis generally depends on the suppression of PI3K/AKT and MAPK survival signaling, induction of pro-apoptotic BCL-2 homology 3 (BH3)-only proteins such as BCL-2-interacting mediator of cell death (BIM), and subsequent activation of BAX and BAK. During prolonged TKI exposure, however, surviving cells may reactivate PI3K/AKT, MAPK, or signal transducer and activator of transcription 3 (STAT3) signaling, maintain the expression of anti-apoptotic proteins such as B-cell lymphoma 2 (BCL-2), B-cell lymphoma-extra large (BCL-xL), and myeloid cell leukemia 1 (MCL-1), or attenuate BIM-mediated apoptosis (Chipuk et al., 2010; Hu et al., 2024; Yang et al., 2022). These changes raise the apoptotic threshold and allow a drug-tolerant population to persist despite continued inhibition of the primary oncogenic driver. Enhanced resistance to apoptosis should therefore be regarded not only as a pre-existing tumor-cell phenotype but also as an adaptive state that can be reinforced by TKI treatment. Preclinical studies of osimertinib resistance further implicate BCL-2/BCL-xL- and MCL-1-dependent survival (Lu et al., 2021; Ma et al., 2022).

Repeated EGFR-TKI exposure may induce tumor-cell populations with increased expression of ATP-binding cassette (ABC) transporters, particularly ABCB1 and ABCG2. For EGFR-TKIs that are transporter substrates, enhanced efflux reduces intracellular drug accumulation and limits sustained target inhibition (Gottesman et al., 2002; Sajid et al., 2023). This effect is drug-dependent because individual EGFR-TKIs may function as substrates, inhibitors, or both. ABCG2 upregulation has been experimentally linked to acquired gefitinib resistance (Chen et al., 2011).

### 2.2 TME-mediated resistance mechanisms

While tumor-intrinsic mechanisms have been extensively studied, they cannot fully account for the complexity and heterogeneity of TKI resistance observed in clinical settings. Increasing evidence indicates that the TME acts as a dynamic and interactive “soil” that supports tumor survival and drives therapeutic resistance through non-cell-autonomous mechanisms. Rather than functioning as passive surrounding tissues, stromal cells, immune cells, extracellular matrix components, and metabolic factors collectively establish a highly adaptive ecosystem that promotes tumor persistence under therapeutic pressure (Binnewies et al., 2018).

#### 2.2.1 Immunosuppressive microenvironment

The antitumor efficacy of EGFR-TKIs is not solely dependent on direct inhibition of oncogenic signaling pathways but also relies heavily on effective immune-mediated elimination of tumor cells. However, the TME frequently establishes a profoundly immunosuppressive ecosystem that limits antitumor immunity and promotes therapeutic resistance.

Across solid tumors, tumor-associated macrophages (TAMs) are among the most abundant immune cell populations within the TME and play a central role in EGFR-TKI resistance. Under tumor-derived cytokines and hypoxic stress, macrophages may acquire M2-like states that secrete interleukin-10 (IL-10) and TGF-β, express programmed death ligand 1 (PD-L1), and recruit regulatory T cells (Tregs), thereby suppressing CD8^+^ cytotoxic T-cell activity (Xiang et al., 2021; Mantovani et al., 2022).

Myeloid-derived suppressor cells (MDSCs) and Tregs are additional immunosuppressive populations described across tumor types. MDSCs can deplete arginine and cysteine, produce reactive oxygen species, and activate suppressive signaling. Tregs further reinforce this immunosuppressive network by directly inhibiting effector T-cell activation and maintaining immune tolerance (Veglia et al., 2021; Facciabene et al., 2012).

Hypoxia-induced glycolysis leads to excessive lactate accumulation, which functions not merely as a metabolic byproduct but also as an immunoregulatory metabolite. Recent evidence suggests that lactate-mediated histone lactylation may promote polarization of TAMs toward the M2 phenotype, thereby establishing a feed-forward loop between metabolic adaptation and immune suppression. Furthermore, lactate accumulation acidifies the extracellular microenvironment, impairs cytotoxic T-cell function, and reduces natural killer (NK) cell activity (Chen et al., 2025; Zhang et al., 2023).

Hypoxia also links metabolic adaptation to immune escape. Hypoxia-inducible factor 1α (HIF-1α) can increase PD-L1 expression in hypoxic tumor cells (Ding et al., 2021). In EGFR-mutant NSCLC, EGFR-TKI exposure has been associated with dynamic changes in PD-L1 expression and the immune microenvironment (Isomoto et al., 2020). These observations support a potential HIF-1α–PD-L1 connection, but its causal contribution to EGFR-TKI resistance remains to be established.

Collectively, these findings indicate that immune suppression in EGFR-TKI-resistant tumors is driven by a highly interconnected immune-metabolic network involving TAMs, MDSCs, Tregs, hypoxia, and lactate signaling.

#### 2.2.2 CAFs, ECM remodeling, and paracrine signaling networks

CAFs are among the most abundant stromal cell populations in the TME and play multifaceted roles in therapeutic resistance. Initially regarded primarily as structural support cells, CAFs are now recognized as active regulators of tumor progression, mechanotransduction signaling, metabolic adaptation, and immune suppression (Biffi and Tuveson, 2021).

One important function of CAFs is ECM remodeling. CAFs secrete excessive amounts of collagen, fibronectin, hyaluronic acid, and other ECM components, resulting in increased tissue stiffness and elevated interstitial fluid pressure. These changes may reduce perfusion and hinder penetration of TKIs into tumor tissues (Biffi and Tuveson, 2021; Kennel et al., 2023; Chen and Song, 2019).

ECM remodeling contributes to resistance not only through physical obstruction but also via biochemical and mechanical signaling. Increased ECM stiffness activates integrin/focal adhesion kinase (FAK) signaling and downstream Yes-associated protein/transcriptional coactivator with PDZ-binding motif (YAP/TAZ) transcriptional programs. In EGFR-mutant NSCLC models, Src family kinase (SFK)/FAK signaling attenuated osimertinib efficacy (Ichihara et al., 2017), whereas integrin-FAK-Src-mediated YAP/TAZ activation has been demonstrated in other tumor models (Ma et al., 2020). Together, these pathways can promote EMT, stemness-associated phenotypes, and survival signaling.

Beyond ECM remodeling, CAFs actively mediate stromal-derived bypass signaling through secretion of cytokines, chemokines, and growth factors. Soluble mediators such as IL-6, hepatocyte growth factor (HGF), C-X-C motif chemokine ligand 12 (CXCL12), and TGF-β activate compensatory pathways including STAT3, PI3K/AKT, MAPK, and NF-κB signaling, thereby sustaining tumor cell survival independently of EGFR inhibition (Thuya et al., 2025; Hu et al., 2021; Fontana et al., 2024; Wei et al., 2026). A related stromal bypass mechanism was demonstrated in BRAF-mutant melanoma, where stromal HGF secretion activated MET and downstream MAPK and PI3K/AKT signaling, conferring innate resistance to RAF inhibitors (Straussman et al., 2012).

In addition, CAF-derived cytokines can induce EMT and promote acquisition of cancer stem cell–like properties. Crosstalk between CAFs and immune cells further amplifies resistance, as CAF-derived TGF-β and CXCL12 contribute to recruitment of immunosuppressive macrophages and Tregs (Hu et al., 2021).

Taken together, CAFs function not merely as passive stromal barriers but as central regulators of the resistant TME through coordinated regulation of ECM remodeling, mechanotransduction, bypass signaling, and immune modulation.

#### 2.2.3 Metabolic reprogramming and hypoxia

Metabolic reprogramming is a hallmark of cancer progression and represents a critical adaptive mechanism underlying resistance to EGFR-TKIs. Under therapeutic pressure and nutrient-deprived conditions, tumor cells undergo profound metabolic alterations that enable sustained proliferation, survival, and immune evasion.

Rapid tumor growth and abnormal vascular architecture frequently result in insufficient oxygen supply, leading to the establishment of hypoxic niches within tumors. Hypoxia stabilizes HIF-1α, a master transcriptional regulator that orchestrates multiple adaptive responses. Activation of HIF-1α promotes glycolytic metabolism through upregulation of glucose transporters and glycolytic enzymes, thereby enhancing glucose uptake and lactate production (Liu et al., 2025; DeBerardinis and Chandel, 2016).

Enhanced aerobic glycolysis reshapes the TME through excessive lactate accumulation, extracellular acidification, and suppression of immune effector function. Lactate also promotes M2 polarization of TAMs and may contribute to epigenetic remodeling through histone lactylation, thereby reinforcing immune escape and therapeutic resistance (Chen et al., 2025; Zhang et al., 2023).

Hypoxia further contributes to resistance through induction of angiogenic and survival signaling pathways. HIF-1α upregulates vascular endothelial growth factor (VEGF) expression, leading to abnormal vascular remodeling characterized by tortuous and hyperpermeable vessels. Although angiogenesis initially improves nutrient supply, the resulting vascular abnormalities paradoxically exacerbate hypoxia and impair drug delivery (Liu et al., 2025).

Furthermore, hypoxia promotes EMT, acquisition of stem-like properties, and activation of survival pathways including PI3K/AKT/mTOR and MAPK signaling. In EGFR-mutant NSCLC, HIF-1α-associated signaling and prolonged hypoxia have been linked to reduced TKI-induced apoptosis and fibroblast growth factor receptor 1 (FGFR1)-MAPK-mediated resistance (Meng et al., 2018; Lu et al., 2020; Jin et al., 2021).

Importantly, metabolic reprogramming is tightly interconnected with stromal remodeling and immune suppression. CAF-mediated ECM stiffening aggravates hypoxia by compressing tumor vasculature, whereas hypoxia-induced cytokines promote CAF activation and inflammatory signaling. These reciprocal interactions collectively establish a highly adaptive metabolic-stromal-immune resistance network (Li et al., 2021).

### 2.3 Integrated TME resistance network

Although immune suppression, extracellular matrix remodeling, paracrine signaling, hypoxia, and metabolic reprogramming are often discussed separately, increasing evidence indicates that these mechanisms form a highly interconnected adaptive network within the TME. Rather than functioning as isolated pathways, these components interact dynamically to establish a self-reinforcing resistant ecosystem that sustains tumor survival under therapeutic pressure (Binnewies et al., 2018).

Reciprocal crosstalk between CAFs, hypoxia, and immune cells appears particularly important. CAF-mediated ECM deposition increases tissue stiffness and compresses tumor vasculature, thereby aggravating hypoxia and impairing drug delivery. In turn, hypoxia promotes activation of CAFs through HIF-1α-dependent signaling, further enhancing ECM and inflammatory cytokine production (Biffi and Tuveson, 2021; Kennel et al., 2023; Chen and Song, 2019; Zhang et al., 2021).

Hypoxia-driven metabolic adaptation also closely interacts with immune suppression. Enhanced glycolysis and lactate accumulation promote M2 polarization of TAMs and suppress cytotoxic T-cell activity (Chen et al., 2025; Zhang et al., 2023; Jin et al., 2025), while HIF-1α-induced PD-L1 expression contributes to immune exhaustion. Simultaneously, CAF-derived cytokines such as IL-6, CXCL12, and TGF-β recruit immunosuppressive immune cells and activate compensatory survival pathways including STAT3, PI3K/AKT, and MAPK signaling (Hu et al., 2021; Fontana et al., 2024; Wei et al., 2026).

Within this network, HIF-1α and TGF-β function as regulatory hubs rather than isolated mediators. Under hypoxia, reduced prolyl hydroxylase activity prevents von Hippel–Lindau (VHL)-dependent degradation, allowing HIF-1α to accumulate and activate its transcriptional program. In EGFR-mutant NSCLC, sustained HIF-1α signaling reduces TKI-induced apoptosis, whereas prolonged hypoxia engages FGFR1-MAPK signaling, attenuates BIM induction, and produces osimertinib resistance (Meng et al., 2018; Lu et al., 2020). High HIF-1α expression has also been associated with acquired EGFR-TKI resistance in NSCLC tissues (Jin et al., 2021). In the surrounding stroma, HIF-1α is required for the activation and tumor-promoting activity of lung CAFs (Zhang et al., 2021). Together with its established effects on glycolysis and VEGF, and its reported relationship with PD-L1 under hypoxia in other tumor models, these findings place HIF-1α at the intersection of metabolic adaptation, bypass signaling, stromal remodeling, and immune escape. (Ding et al., 2021; Liu et al., 2025; DeBerardinis and Chandel, 2016), TGF-β forms a complementary stromal-plasticity hub. Produced by tumor cells and CAFs, TGF-β activates canonical SMAD2/3 signaling and, depending on cellular context, noncanonical pathways including NF-κB, PI3K/AKT, and MAPK. These outputs reinforce CAF activation and ECM deposition, drive EMT and drug-tolerant cell states, and contribute to T-cell exclusion and suppression (Biffi and Tuveson, 2021; Kennel et al., 2023; Chen and Song, 2019). Direct evidence in EGFR-mutant NSCLC shows that osimertinib can induce TGF-β2, leading to SMAD2-dependent EMT and NF-κB-dependent survival in resistant cells (Jiang et al., 2021). The two hubs also reinforce one another at the tissue level: HIF-1α promotes fibroblast activation, whereas TGF-β-driven stromal remodeling can compress vessels and deepen hypoxia.

**FIGURE 1:**
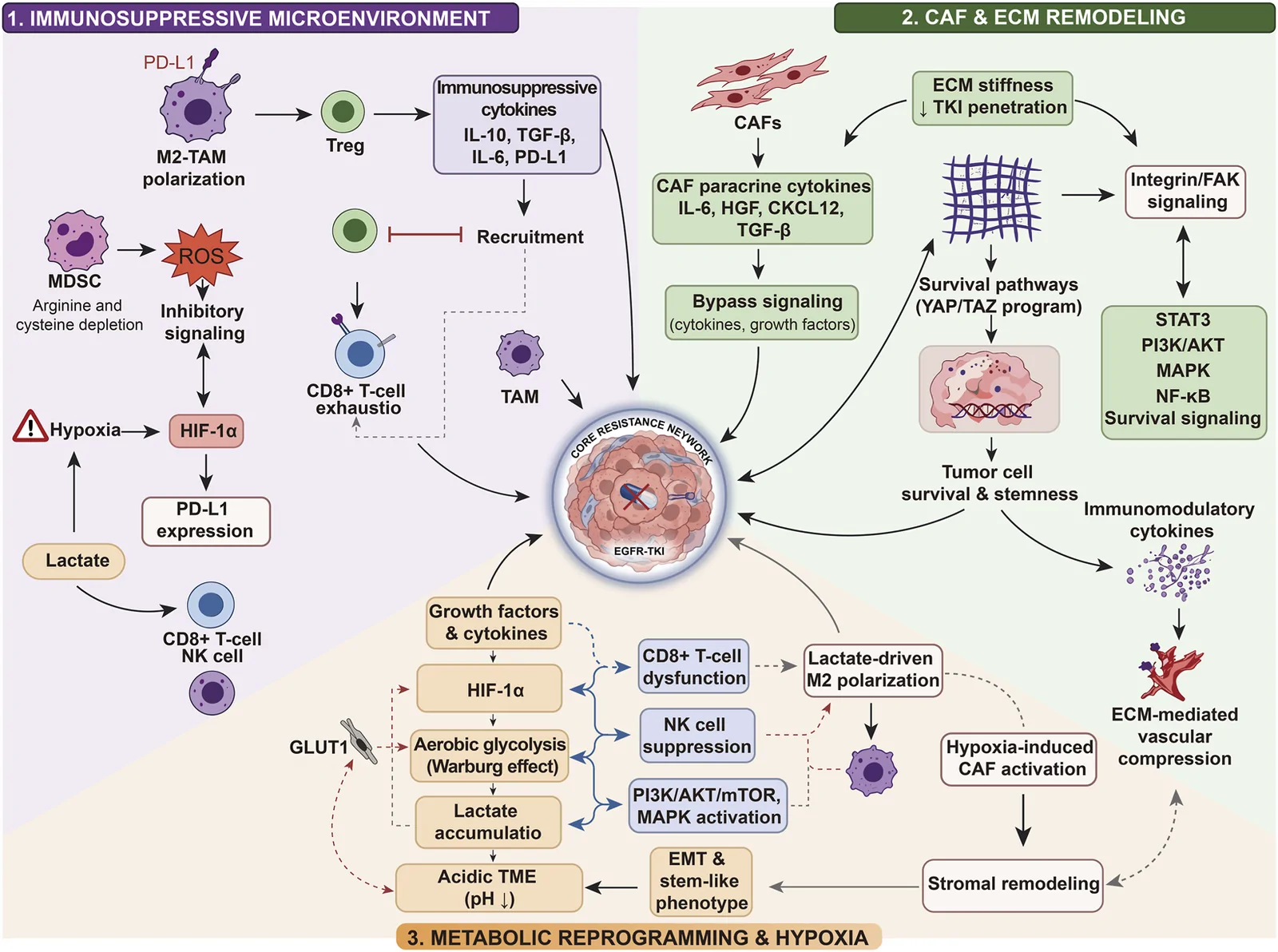
Integrated TME-mediated network of EGFR-TKI resistance in NSCLC.

These interconnected processes may create a feed-forward resistance circuitry characterized by persistent inflammation, immune escape, stromal remodeling, metabolic adaptation, and survival signaling. Figure 1 summarizes this proposed network specifically for EGFR-TKI resistance in NSCLC.

This systems-level perspective provides a rationale for testing TCM-based adjuncts with EGFR-TKIs. TCM, characterized by multi-target and multi-pathway activities, may simultaneously regulate immune responses, stromal activation, angiogenesis, metabolic adaptation, and inflammatory signaling. Therefore, TCM-based interventions have the potential to reprogram the resistant tumor ecosystem and restore TKI sensitivity at the systems level (Liu et al., 2024; Qi et al., 2025; Wang Z. et al., 2024).

## 3 TCM-derived strategies targeting tumor-intrinsic and TME-mediated TKI resistance

TKI resistance is sustained by both tumor-intrinsic alterations and a highly complex TME (Passaro et al., 2021; Binnewies et al., 2018). Inhibition of a single oncogenic driver can select resistant clones, activate bypass signaling, and induce non-genetic cell states; immune, stromal, vascular, and metabolic factors can further stabilize these adaptations.

TCM, characterized by multi-component and multi-target properties, offers a different therapeutic paradigm. Its constituents may act directly on resistant tumor cells while also modulating immune, stromal, vascular, and metabolic components of the TME. This breadth should not, however, be equated with proven systems-level activity. The chemical composition and systemic exposure of many preparations remain incompletely characterized, and findings from an isolated compound cannot be extrapolated to a complex formula or to clinically used doses (Liu et al., 2024; Wang Z. et al., 2024; Zhang et al., 2018). Mechanistic and clinical evidence are therefore considered separately.

This section first examines evidence that Chinese medicine-derived compounds or preparations directly re-sensitize resistant NSCLC cells, and then considers their effects on immune, stromal, vascular, and metabolic components of the TME.

### 3.1 Direct targeting of tumor-intrinsic TKI-resistance mechanisms

Several TCM-derived compounds have restored TKI response by acting directly on resistant NSCLC cells, although the evidence remains preclinical. Costunolide inhibited mitogen-activated protein kinase kinase 1 (MEK1) and AKT1/2 and enhanced osimertinib activity in resistant cell lines and an osimertinib-resistant patient-derived xenograft model (Tian et al., 2022). Honokiol promoted MCL-1 degradation and increased osimertinib-induced apoptosis in resistant cells and xenografts harboring EGFR exon 19 deletion/T790M/C797S (Zang et al., 2020).

These studies show functional re-sensitization through inhibition of bypass survival signaling or apoptosis evasion; they do not eliminate the underlying resistance mutation. No corresponding mechanism has yet been validated in patients. These agents should therefore be regarded as preclinical sensitizers rather than established resistance-reversal therapies.

### 3.2 Reprogramming the tumor immune microenvironment

The immunosuppressive microenvironment is a major barrier to effective TKI therapy, because it limits immune-mediated tumor clearance. TCM-derived compounds have demonstrated significant potential in restoring immune function and enhancing anti-tumor immunity.

Astragalus polysaccharides (APS) are among the most extensively studied TCM components with immunomodulatory activity. In a preclinical study, the injectable APS preparation PG2 promoted M1-like macrophage polarization, dendritic-cell maturation, and T-cell-mediated antitumor responses in NSCLC cells, patient-derived samples, and a lung-tumor model (Bamodu et al., 2019).

Experimental macrophage studies have linked APS-mediated immunomodulation to calcium ion (Ca2+)/cyclic adenosine monophosphate (cAMP) and Toll-like receptor 4 (TLR4)/NF-κB signaling (Wang et al., 2017). One TKI-specific cell study provides a more direct link. In gefitinib-resistant PC9-GR and H1975 cells, Shenqi Fuzheng injection reduced IL-22 expression and STAT3/AKT phosphorylation and restored gefitinib-associated growth inhibition and apoptosis (Wang J et al., 2024).

At the patient level, a retrospective cohort of EGFR-mutant NSCLC associated Shenqi Fuzheng injection plus first-generation EGFR-TKIs with longer progression-free survival (PFS) and fewer adverse events than EGFR-TKI alone (Wang J. L. et al., 2023). The study provides preliminary clinical outcome evidence.

Ginsenoside Rg3 has been investigated as a potential adjunct to EGFR-TKIs in NSCLC, with emerging evidence suggesting that it may reinforce EGFR blockade at both receptor and resistance-state levels. In EGFR-mutant HCC827 lung adenocarcinoma cells, Rg3 was reported to reduce copy-number signals across EGFR exons 18–21 and decrease EGFR protein abundance; complementary antitumor activity was also observed when Rg3 and gefitinib were administered sequentially in a mouse lung tumor model (Lv X et al., 2025). Earlier experiments in A549 and H1299 cells showed that Rg3 enhanced gefitinib-induced growth inhibition and apoptosis, accompanied by increased BAX and cleaved caspase-3, decreased BCL-2, and partial reversal of the migratory phenotype through downregulation of Snail and Slug and restoration of E-cadherin (Dai et al., 2019). In osimertinib-resistant H1975 cells, Rg3 additionally suppressed YAP/TAZ-dependent stemness through activation of Hippo signaling, indicating that its sensitizing activity may extend beyond direct regulation of EGFR abundance (Tan et al., 2020).

Consistent with these preclinical observations, a three-center retrospective cohort of 124 patients with advanced EGFR-mutant NSCLC found that the addition of Rg3 to first-generation EGFR-TKIs was associated with longer median progression-free survival (12.4 vs. 9.9 months) and a higher objective response rate (ORR; 59.6% vs. 41.7%), without a significant overall survival (OS) benefit or an apparent increase in grade ≥2 toxicity (Li et al., 2016). However, the clinical study was non-randomized, had an imbalance in the EGFR-TKIs administered between groups, and did not include serial tumor sampling or molecular analyses to confirm modulation of EGFR or resistance pathways. Moreover, the reported reduction in EGFR copy number is based on a recent preclinical study and requires independent validation. Rg3 should therefore be considered a biologically plausible but clinically exploratory EGFR-TKI sensitizer rather than an established treatment for EGFR-mutant NSCLC.

Collectively, these findings suggest that TCM-mediated immune reprogramming may help overcome TKI resistance, although direct clinical evidence remains limited.

### 3.3 Targeting cancer-associated fibroblasts and stromal barriers

CAFs and the ECM they produce are key structural components of the TME that contribute to drug resistance by limiting drug penetration and providing survival signals to tumor cells.

Curcumin has been proposed to modulate stromal barriers; however, direct evidence that it acts on CAFs or improves TKI penetration in NSCLC remains limited. In A549 and PC-9 lung cancer cells, curcumin inhibited HGF-induced MET/PI3K/AKT/mTOR signaling, EMT, and angiogenic activity (Jiao et al., 2016); however, the study did not test CAFs or EGFR-TKI resistance.

In addition, curcumin exhibits anti-inflammatory and immunomodulatory properties, further contributing to its therapeutic potential (Wang Z. et al., 2024).

Curcumin also enhanced gefitinib activity in primary-resistant H157 and H1299 NSCLC cells and xenografts through autophagy-related cell death and specificity protein 1 (Sp1)/EGFR downregulation (Chen et al., 2019).

Thus, curcumin provides evidence of direct tumor-cell sensitization, whereas its ability to inhibit CAF-mediated extracellular-matrix barriers or improve TKI delivery remains unconfirmed.

### 3.4 Inhibition of pathological angiogenesis and vascular normalization

Abnormal tumor vasculature contributes to hypoxia, impairs drug delivery, and facilitates resistance. Some TCM preparations have been proposed to inhibit pathological angiogenesis; however, evidence for true vascular normalization, defined by improved perfusion and oxygenation, remains limited (Shen et al., 2013).

In a Lewis lung carcinoma model, Kanglaite combined with gefitinib reduced tumor microvessel formation and VEGF/kinase insert domain receptor (KDR) expression (Shen et al., 2013). This supports an antiangiogenic effect in a murine lung-tumor model, but the study did not measure pericyte coverage, tumor perfusion, oxygenation, or intratumoral gefitinib exposure. It therefore does not establish vascular normalization.

A meta-analysis of 12 trials described as randomized, involving 1,046 patients with stage III/IV NSCLC, reported higher disease control rate (odds ratio (OR), 3.26; 95% confidence interval (CI), 2.22–4.77) and objective response rate (OR, 2.59, 95% CI 1.87–3.58) with Kanglaite plus a first-generation EGFR-TKI than with the TKI alone (Kong et al., 2021).

Kanglaite is a clinically investigated adjunct with low-certainty, short-term response data. Its antiangiogenic mechanism is supported by a murine lung-cancer study, whereas vascular normalization and delayed EGFR-TKI resistance remain unverified hypotheses.

### 3.5 Correction of metabolic dysregulation in the TME

Metabolic reprogramming, particularly hypoxia and extracellular acidosis, is a major driver of TKI resistance. Some TCM-derived compounds have been reported to modulate tumor metabolism and restore metabolic balance within the TME (Wang et al., 2021).

A recent study in the A549 and H1299 NSCLC cell lines showed that berberine reduced glucose consumption, lactate production, and the ATP/ADP ratio through KIF20A/CCNE2-dependent inhibition of PI3K/AKT signaling (Wang Q. et al., 2023). In A549 and H1299 cells and xenografts, berberine also inhibited growth, invasion, and glycolysis through the SPC25/NUF2 axis (Lv M. et al., 2025).

Andrographolide reduced HIF-1α and VEGF expression in hypoxic A549 NSCLC cells (Lin et al., 2011). A separate study in A549, H292, and H522 NSCLC cells linked reduced lactate production and increased mitochondrial respiration to downregulation of pyruvate dehydrogenase kinase 1 (Yang et al., 2023). These cell-based findings support interference with hypoxia-responsive metabolism but do not establish restoration of TKI sensitivity.

These studies identify candidate tumor-cell metabolic mechanisms for berberine and andrographolide. They do not yet show clinical benefit or demonstrate reversal of TME acidosis or hypoxia, restoration of antitumor immunity, or prolongation of EGFR-TKI response. These compounds should therefore be presented as preclinical leads rather than validated TME-targeted sensitizers.

**FIGURE 2:**
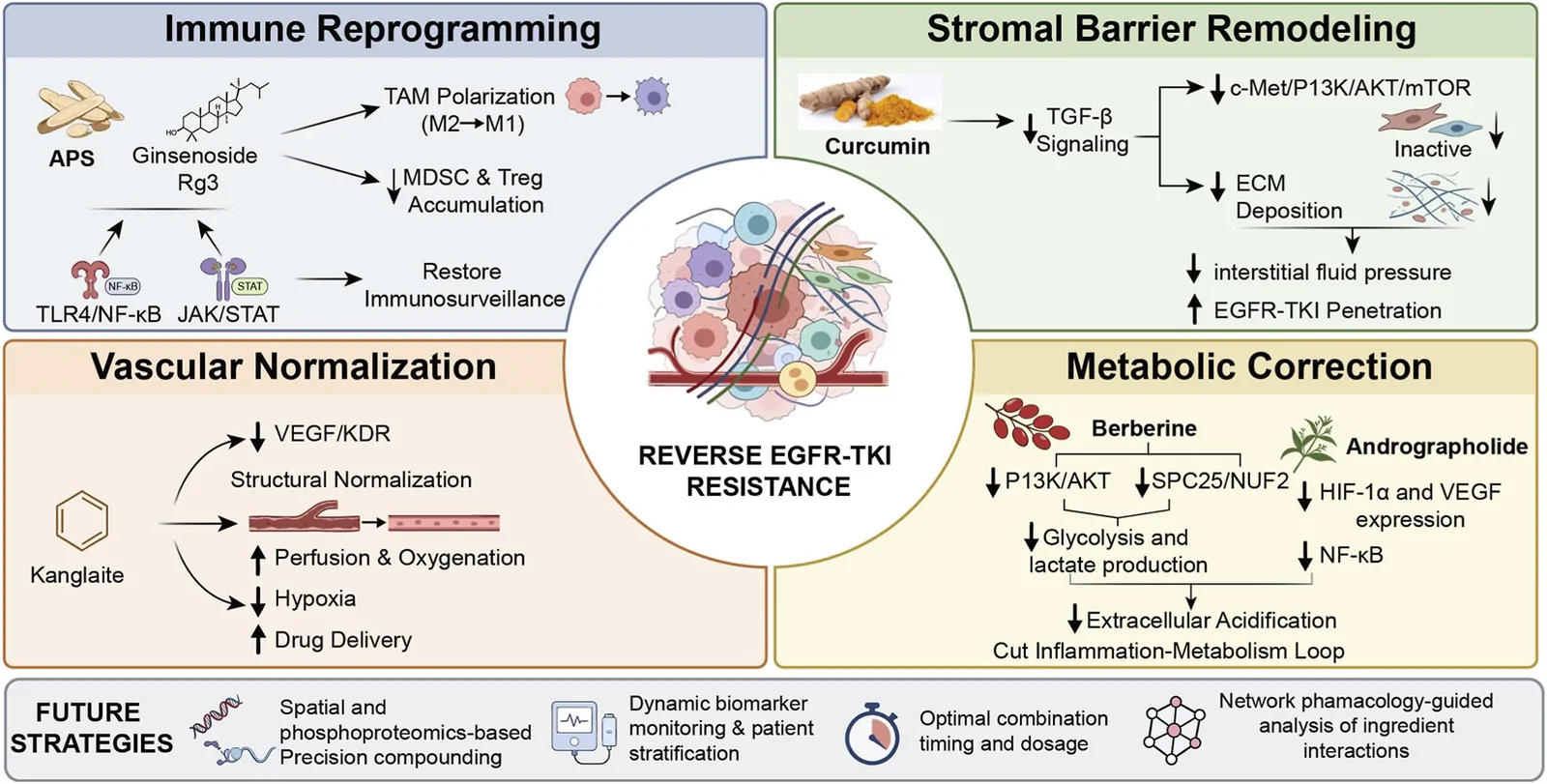
TCM-derived strategies for reprogramming the TME and potential attenuation of EGFR-TKI resistance.

Taken together, Chinese medicine-derived agents may act directly on resistant tumor cells and indirectly through immune, stromal, vascular, and metabolic pathways. Figure 2 summarizes these representative mechanisms. However, the evidence remains uneven: clinical support is limited mainly to short-term outcomes from heterogeneous adjunctive studies, whereas most mechanistic claims derive from cell and animal models. This distinction should be maintained when considering these agents as adjuncts to TKIs.

To facilitate comparison across heterogeneous experimental designs, the preparation, dose, and administration route of representative Chinese medicine-derived interventions discussed in this section are summarized in Table 1.

**TABLE 1:** Representative study conditions and evidence status of Chinese medicine-derived interventions.

Chinese medicine	Study model	Dose/regimen	Preparation and administration	Principal observation and evidence status
Costunolide (Tian et al., 2022)	Osimertinib-resistant EGFR-mutant NSCLC cells and resistant PDX model	Cells: 10 μM with osimertinib 1 μM; PDX: 20 mg/kg with osimertinib 10 mg/kg, q.d	Isolated sesquiterpene lactone; oral administration in the PDX model	MEK1 and AKT1/2 inhibition; restored osimertinib response; Exploratory (preclinical)
Honokiol (Zang et al., 2020)	Osimertinib-resistant EGFR-mutant NSCLC cells and xenografts	Cells: 10 μM with osimertinib 1 μM; mice: 50 mg/kg/day with osimertinib 10 mg/kg/day	Isolated biphenolic compound; honokiol i.p. and osimertinib orally, 5 days/week	MCL-1 degradation and increased apoptosis Exploratory (preclinical)
Astragalus polysaccharide PG2 (Bamodu et al., 2019)	H441/H1299 cells, human NSCLC samples and a lung-tumor mouse model	Cells: 16 mg/mL; mice: 3 mg/kg/day	Lyophilized injectable polysaccharide preparation reconstituted in saline; experimental administration route NR	Macrophage, dendritic-cell, and T-cell immune changes; Exploratory (preclinical)
Shenqi Fuzheng injection (Wang J. L. et al., 2023)	Retrospective cohort of advanced EGFR-mutant NSCLC, n = 88	250 mL q.d. for 21 days	Commercial Codonopsis–Astragalus injection; i.v	Association with longer PFS and fewer adverse events Exploratory clinical, retrospective
Curcumin (Chen et al., 2019)	Gefitinib-resistant H157/H1299 cells and xenografts	Cells: 10 μM with gefitinib 5 μM for 48 h; mice: 1 g/kg with gefitinib 100 mg/kg, q.d	Isolated compound, propylene-glycol vehicle; oral gavage	Autophagy-related cell death and Sp1/EGFR downregulation Exploratory (preclinical)
Kanglaite (Kong et al., 2021)	Twelve studies, n = 1,046, involving stage III–IV NSCLC and first-generation EGFR-TKIs	Injection: 100–200 mL/day; capsules: 2.7 g q.i.d.; generally 3–9 weeks or 60 days	Commercial coix-seed-oil injection in 10 studies and capsules in 2	Higher short-term ORR and DCR, without survival confirmation; Exploratory (clinical)
Berberine (Wang Q. et al., 2023; Lv M et al., 2025)	A549/H1299 cells and xenografts	Cells: 20–80 μM (Wang Q. et al., 2023)	Isolated isoquinoline alkaloid; cell exposure	Reduced glycolysis through KIF20A/CCNE2 and SPC25/NUF2 signaling Exploratory (preclinical)
Andrographolide (Lin et al., 2011; Yang et al., 2023)	A549/H292/H522 cells	10–50 μM (Lin et al., 2011); 20–75 μM for 6–24 h (Yang et al., 2023)	Isolated diterpene lactone; cell exposure	Reduced glycolysis, HIF-1α signaling, and PDK1 expression Exploratory (preclinical)

Data are representative rather than exhaustive. NR, not reported; i.p., intraperitoneal; i.v., intravenous; PDX, patient-derived xenograft; q.d., once daily; q.i.d., four times daily.

## 4 Challenges and future perspectives

### 4.1 Major challenges in TCM-based strategies for overcoming TKI resistance

Despite the promising potential of TCM in overcoming TKI resistance, several critical challenges remain that limit its clinical translation and widespread adoption (Naeem et al., 2022).

#### 4.1.1 Ambiguity in active components and mechanisms

One of the most significant challenges lies in the unclear pharmacological basis of TCM formulations. Unlike conventional drugs that typically target a single molecule or pathway, TCM prescriptions consist of multiple bioactive compounds with complex and often overlapping functions. While this multi-component nature provides theoretical advantages for systems-level regulation, it also complicates mechanistic studies.

It remains difficult to precisely identify the key active components responsible for therapeutic effects, as well as to quantify their individual contributions and potential synergistic or antagonistic interactions. Current approaches, such as network pharmacology and systems biology, have provided valuable insights but are still insufficient to fully resolve these complexities. This lack of mechanistic clarity represents a major barrier to the standardization and scientific validation of TCM.

#### 4.1.2 Variability and quality control issues

Another major limitation is the variability in TCM preparations. The chemical composition of herbal medicines can be influenced by multiple factors, including geographical origin, cultivation conditions, harvesting time, and processing methods. These variations lead to inconsistencies in pharmacological activity and therapeutic efficacy.

Moreover, standardized quality control systems for TCM are not yet universally established. The absence of robust quality assurance frameworks not only affects reproducibility in preclinical studies but also limits the reliability of clinical outcomes. Developing standardized protocols for raw material sourcing, processing, and quality evaluation is therefore essential for advancing TCM research.

#### 4.1.3 Insufficient high-quality clinical evidence

Although numerous studies have demonstrated the potential of TCM in enhancing TKI efficacy, the majority of these studies are limited by small sample sizes, retrospective designs, or single-center settings. High-quality evidence from large-scale, randomized, double-blind, multicenter clinical trials remains scarce (Wang J. L. et al., 2023; Kong et al., 2021; Li et al., 2016).

Patient-level evidence is limited to retrospective cohorts and meta-analyses of trials with substantial reporting limitations. The Shenqi Fuzheng and Rg3 studies were retrospective (Wang J. L. et al., 2023; Li et al., 2016). In the Kanglaite meta-analysis, no trial reported PFS or OS; allocation concealment was uncommon; and blinding was absent (Kong et al., 2021). These designs may generate useful signals but do not establish efficacy with the certainty expected for guideline adoption.

#### 4.1.4 Complexity of TME interactions

The TME is a highly dynamic and context-dependent system, with complex interactions among immune, stromal, and metabolic components. While preclinical studies have demonstrated that TCM can modulate multiple aspects of the TME, translating these findings into clinical applications remains challenging.

Differences between experimental models and human tumors, as well as interpatient heterogeneity, may lead to discrepancies in therapeutic outcomes. Furthermore, the lack of predictive biomarkers makes it difficult to identify patients who are most likely to benefit from TCM-based interventions.

Although EGFR-mutant NSCLC provides a well-characterized model of targeted-therapy resistance, its resistance biology is not necessarily interchangeable with that of other RTK-driven tumors. The applicability of TME-targeted TCM strategies should therefore be validated within each molecular and therapeutic context.

### 4.2 Future research directions

To fully realize the therapeutic potential of TCM in overcoming TKI resistance, future research should focus on integrating advanced technologies, improving clinical study design, and developing precision medicine approaches.

#### 4.2.1 Network pharmacology-guided analysis of ingredient interactions

A useful framework should begin with chemistry rather than database connectivity. Each batch should undergo chromatographic and high-resolution mass-spectrometric fingerprinting with quantitative measurement of major constituents; candidate components should then be filtered by plasma or tumor exposure at clinically relevant doses. Network pharmacology can integrate exposed constituents with transcriptomic, proteomic, and metabolomic changes in EGFR-TKI-resistant tumor cells, TAMs, CAFs, endothelial cells, and other TME compartments to construct an ingredient-target-cell type-resistance phenotype network (Zhao et al., 2023; Li and Xiao, 2025). Within this network, a candidate principal component would act on the dominant resistance driver or restore EGFR-TKI sensitivity, whereas a candidate adjuvant component would provide a complementary effect on immune suppression, including TAM polarization (Qi et al., 2025), stromal signaling, hypoxia or metabolism, drug penetration, or treatment toxicity. This assignment is operational and should not be inferred from database connectivity or traditional hierarchy alone.

Predicted pairs should be tested in full dose-response matrices that include each constituent alone (Foucquier and Guedj, 2015; Ianevski et al., 2022). Results should then be replicated in EGFR-TKI-resistant co-cultures, organoids, and *in vivo* models with TME readouts. Genetic or pharmacological perturbation of the nominated hub should abolish the interaction, while pharmacokinetic measurements should determine whether the apparent benefit reflects altered EGFR-TKI exposure rather than pharmacodynamic cooperation. Only combinations showing reproducible supra-additive activity at clinically achievable concentrations without increased toxicity should be described as synergistic. The Jun-Chen-Zuo-Shi framework may guide hypothesis generation, but principal-adjuvant roles should be assigned only after such component-level evidence.

#### 4.2.2 Development of TME-targeted TCM-based therapies

Future studies should focus on developing TCM formulations that specifically target defined components of the TME. For example, therapies could be designed to selectively modulate immune cell subsets, inhibit CAF activation, or normalize tumor vasculature.

The identification of TME-specific biomarkers, such as immune cell signatures or metabolic profiles, will enable more precise targeting and improve therapeutic efficacy. This approach represents a shift from empirical treatment toward mechanism-driven drug development.

#### 4.2.3 Exploration of emerging regulatory axes, including the gut–tumor axis

Recent studies have highlighted the role of the gut microbiota in shaping systemic immunity and influencing tumor progression. The gut–tumor axis has emerged as a novel regulatory pathway with significant therapeutic potential.

TCM-microbiota interactions are an emerging research direction, but their relevance to EGFR-TKI resistance in NSCLC has not been established. Future studies should determine whether changes in microbiota composition or microbial metabolites are reproducibly linked to systemic immunity, tumor infiltration, and treatment response.

Further exploration of these interactions may uncover new mechanisms by which TCM enhances antitumor responses and overcomes resistance.

#### 4.2.4 Optimization of combination therapy strategies

Combining TCM with conventional therapies represents a promising strategy for improving treatment outcomes. Future studies should focus on optimizing combination regimens, including dosing schedules, treatment duration, and patient selection.

Some adjunctive studies have reported fewer adverse events or improved performance status with TCM preparations plus EGFR-TKIs (Wang J. L. et al., 2023; Kong et al., 2021). These signals require prospective confirmation with standardized products and prespecified safety and quality-of-life endpoints.

#### 4.2.5 Precision medicine and biomarker-driven patient stratification

Because the TME varies across tumor genotype, treatment history, and resistance state, patient selection should be based on prespecified biomarkers rather than on a generic diagnosis. Candidate measures include immune-cell composition, CAF/ECM signatures, hypoxia or metabolic profiles, and the tumor-intrinsic resistance alteration targeted by the combination.

An exploratory enrollment panel could combine tumor PD-L1 expression, TAM infiltration (density and spatial phenotype), and intratumoral lactate or a validated hypoxia-glycolysis signature. These variables should be analyzed as a composite rather than used as stand-alone companion diagnostics. PD-L1 can change during EGFR-TKI exposure (Isomoto et al., 2020), TAMs are phenotypically diverse and treatment-responsive (Pittet et al., 2022), and lactate has context-dependent effects on immune-cell function (Certo et al., 2021). A biomarker-enrichment study could therefore evaluate TCM candidates with reproducible immune-modulating activity in PD-L1-high/TAM-rich tumors, and candidates with reproducible metabolic effects in lactate-high or hypoxic tumors. Assays and cut-offs should be prespecified, paired baseline and on-treatment biopsies should confirm target engagement, and randomized cohorts should test a treatment-by-biomarker interaction. Until prospective validation is available, these profiles should be regarded as exploratory selection hypotheses rather than indications for clinical use.

Future development should pair baseline and on-treatment sampling with pharmacokinetic and exposure-response analyses. Candidate combinations should progress from standardized product characterization to EGFR-TKI-resistant TME-competent models, early trials with paired tissue or circulating biomarkers, and finally biomarker-stratified randomized trials powered for PFS, overall survival, safety, and quality of life.

## 5 Conclusion

An integrated approach to EGFR-TKI resistance must consider both tumor-intrinsic changes and the surrounding tumor ecosystem.

Preclinical evidence suggests that TCM-derived interventions can affect immune suppression, stromal barriers, vascular abnormalities, and metabolic dysregulation. These effects may complement EGFR inhibition, but evidence for systems-level reprogramming in patients remains limited.

However, significant challenges remain, including the need for mechanistic clarification, standardization of formulations, and high-quality clinical validation. Addressing these issues will be essential for translating TCM-based strategies into clinical practice.

Future studies should link standardized chemical characterization and exposure data with TME-competent resistance models, paired biomarker sampling, and adequately powered clinical trials. On current evidence, TCM-based combinations remain investigational but warrant rigorous evaluation in EGFR-TKI-resistant NSCLC.

## Glossary

**ABC**: ATP-binding cassette
ADP: adenosine diphosphate
AKT: Protein kinase B
ALK: anaplastic lymphoma kinase
APS: Astragalus polysaccharides
ATP: Adenosine triphosphate
AXL: AXL receptor tyrosine kinase
BCL-2: B-cell lymphoma 2
BCL-xL: B-cell lymphoma-extra large
BH3: BCL-2 homology 3
BIM: BCL-2-interacting mediator of cell death
CAF: Cancer-associated fibroblast
cAMP: cyclic adenosine monophosphate
CD8: Cluster of differentiation 8
CI: confidence interval
CXCL12: C-X-C motif chemokine ligand 12
DCR: Disease control rate
ECM: Extracellular matrix
EGF: Epidermal growth factor
EGFR: Epidermal growth factor receptor
EGFR-TKI: epidermal growth factor receptor tyrosine kinase inhibitor
EMT: Epithelial-to-mesenchymal transition
ERK: Extracellular signal-regulated kinase
FAK: Focal adhesion kinase
FGFR1: Fibroblast growth factor receptor 1
GLUT1: Glucose transporter 1
HER2: Human epidermal growth factor receptor 2
HGF: Hepatocyte growth factor
HIF-1α: Hypoxia-inducible factor 1α
HK2: Hexokinase 2
IGF-1: Insulin-like growth factor 1
IL: Interleukin
JAK: Janus kinase
KDR: Kinase insert domain receptor
MAPK: Mitogen-activated protein kinase
MCL-1: Myeloid cell leukemia 1
MDSC: Myeloid-derived suppressor cell
MEK1: Mitogen-activated protein kinase kinase 1
MET: Mesenchymal-epithelial transition factor
mTOR: Mechanistic target of rapamycin
NF-κB: nuclear factor kappa-light-chain-enhancer of activated B cells
NK: Natural killer
NSCLC: Non-small cell lung cancer
OR: odds ratio
ORR: Objective response rate
OS: Overall survival
PD: Pharmacodynamics
PD-L1: Programmed death-ligand 1
PFS: Progression-free survival
PI3K: Phosphoinositide 3-kinase
ROS: Reactive oxygen species
RTK: Receptor tyrosine kinase
SFK: Src family kinase
Sp1: Specificity protein 1
STAT: Signal transducer and activator of transcription
TAM: Tumor-associated macrophage
TAZ: Transcriptional coactivator with PDZ-binding motif
TCM: Traditional Chinese medicine
TGF-β: Transforming growth factor beta
TKI: Tyrosine kinase inhibitor
TLR4: Toll-like receptor 4
TME: Tumor microenvironment
TNF-α: Tumor necrosis factor alpha
Treg: Regulatory T cell
VEGF: Vascular endothelial growth factor
VEGFR: Vascular endothelial growth factor receptor
VHL: von Hippel-Lindau
YAP: Yes-associated protein
